# MicrO: an ontology of phenotypic and metabolic characters, assays, and culture media found in prokaryotic taxonomic descriptions

**DOI:** 10.1186/s13326-016-0060-6

**Published:** 2016-04-12

**Authors:** Carrine E. Blank, Hong Cui, Lisa R. Moore, Ramona L. Walls

**Affiliations:** Department of Geosciences, University of Montana, Missoula, MT 59812 USA; School of Information, University of Arizona, Tucson, AZ 85719 USA; Department of Biological Sciences, University of Southern Maine, Portland, ME 04104 USA; CyVerse, University of Arizona, Tucson, AZ 85721 USA

**Keywords:** Prokaryotes, Microbes, Ontology, ChEBI, Gene Ontology, Metabolic characters, Natural language processing, Prokaryotic taxonomy, Bacteria, Archaea, Microbial bioinformatics

## Abstract

**Background:**

MicrO is an ontology of microbiological terms, including prokaryotic qualities and processes, material entities (such as cell components), chemical entities (such as microbiological culture media and medium ingredients), and assays. The ontology was built to support the ongoing development of a natural language processing algorithm, MicroPIE (or, Microbial Phenomics Information Extractor). During the MicroPIE design process, we realized there was a need for a prokaryotic ontology which would capture the evolutionary diversity of phenotypes and metabolic processes across the tree of life, capture the diversity of synonyms and information contained in the taxonomic literature, and relate microbiological entities and processes to terms in a large number of other ontologies, most particularly the Gene Ontology (GO), the Phenotypic Quality Ontology (PATO), and the Chemical Entities of Biological Interest (ChEBI). We thus constructed MicrO to be rich in logical axioms and synonyms gathered from the taxonomic literature.

**Results:**

MicrO currently has ~14550 classes (~2550 of which are new, the remainder being microbiologically-relevant classes imported from other ontologies), connected by ~24,130 logical axioms (5,446 of which are new), and is available at (http://purl.obolibrary.org/obo/MicrO.owl) and on the project website at https://github.com/carrineblank/MicrO. MicrO has been integrated into the OBO Foundry Library (http://www.obofoundry.org/ontology/micro.html), so that other ontologies can borrow and re-use classes. Term requests and user feedback can be made using MicrO’s Issue Tracker in GitHub. We designed MicrO such that it can support the ongoing and future development of algorithms that can leverage the controlled vocabulary and logical inference power provided by the ontology.

**Conclusions:**

By connecting microbial classes with large numbers of chemical entities, material entities, biological processes, molecular functions, and qualities using a dense array of logical axioms, we intend MicrO to be a powerful new tool to increase the computing power of bioinformatics tools such as the automated text mining of prokaryotic taxonomic descriptions using natural language processing. We also intend MicrO to support the development of new bioinformatics tools that aim to develop new connections between microbial phenotypes and genotypes (*i.e.,* the gene content in genomes). Future ontology development will include incorporation of pathogenic phenotypes and prokaryotic habitats.

**Electronic supplementary material:**

The online version of this article (doi:10.1186/s13326-016-0060-6) contains supplementary material, which is available to authorized users.

## Background

Microorganisms comprise most of the evolutionary and genetic diversity in the tree of life [1–3], and produce a significant proportion of the standing crop of cellular carbon on the Earth [4, 5]. Prokaryotic microorganisms manifest their diversity in the form of morphological phenotypes (such as biofilm formation, multicellularity, and differentiation into specialized structures), ecological phenotypes (inhabiting environments that have particular temperature, salinity, and pH values), metabolic phenotypes (the ability to catalyze discrete chemical reactions), and the ability to perform biological processes (carrying out photosynthesis) [6]. Several studies have examined the evolution of microbial phenotypic traits in deep time [7–13]. Nevertheless, most of these studies have focused on relatively small taxonomic groups, or have used a small number of phenotypic traits. This is because the taxon-by-character matrices (which record the presence and absence of traits for each taxon) required for these studies have been constructed manually and thus require significant efforts to build. Hence, the field needs to develop tools that can allow the accelerated, broad-scale study of the evolution of phenotypic traits across the prokaryotic domains of life.

Bioinformatics resources that are needed to accelerate such evolutionary studies include tools that permit the rapid processing of large amounts of legacy text and databases (which contains detailed information on phenotypes and metadata) as well as tools that facilitate the rapid processing of genotypic data (genomic sequences). Such tools could lead to new profound insights in broad-scale microbial evolution, as well as lead to new mechanisms for genome annotation (by associating novel phenotypes with genotypes). To address some of these needs, our team has developed an ontology to assist development of a new natural language processing (NLP) algorithm, MicroPIE (or Microbial Phenomics Information Extractor; https://github.com/biosemantics/micropie2) [14]. MicroPIE is designed to automatically extract text from prokaryotic taxonomic descriptions and to export a character matrix. The character matrix can then be used to study the evolution of traits using phylogenetic comparative methods. Most prokaryotic taxonomic descriptions are published in the International Journal of Systematic and Evolutionary Microbiology (IJSEM) and follow a semi-formalized structure. However, this structure has changed over time, and the content within descriptions (types of reported or assayed phenotypic characters, as well as naming conventions for chemical entities) has also changed. Some taxonomic descriptions (such as for the Cyanobacteria) are usually published outside the IJSEM and have historically followed the botanical code [15, 16], thus they often have different information content. Also, during the development of MicroPIE, we observed that different authors can have markedly different ways of naming or describing synonymous prokaryotic structures and processes (for example they might describe the morphology of rods as an elongated cocci, short cylinders, or bacilli), making NLP treatment of text from taxonomic descriptions challenging.

Presently, some of the text extraction algorithms within MicroPIE use a list of terms that includes all the synonyms we have found in a sampling of the prokaryotic taxonomic literature. However, the term lists treat all synonyms as distinct terms. Also, in prokaryotic taxonomic descriptions there is variability in how common traits are described. For example, we have observed that authors report a positive result of the indole assay as “indole test positive”, “indole-positive”, “indole production”, “indole reaction is positive”, “indole formed”, or “tryptophanase produced”. While a domain-expert would immediately recognize that these are all synonymous, a computer or non-domain expert may not. Finally, NLP supported by term lists lacks inference power. For instance, NLP cannot infer that an organism with an optimal growth temperature of 60 °C is a thermophile.

Through the MicroPIE development process, it became evident that the field needed a robust ontology. While an early version of the Ontology of Microbial Phenotypes (OMP) was available [17, 18], it was focused on *E. coli* phenotypes and had a structure that did not readily lend itself to term re-use. Thus, we created a new ontology, MicrO, which suited our project’s needs and that could be usable by the ontology community at large. This ontology captures much of the evolutionary diversity of prokaryotic traits and processes and the rich legacy of material entity, quality, and assay terms that encompasses the vast diversity found throughout the prokaryotic taxonomic literature. We also designed the ontology to use as a controlled vocabulary that linked the diversity of synonyms found in the literature to central terms that will help support text mining algorithms such as MicroPIE. The ontology leverages logical inference power (for example, to predict that an aerobic microorganism that metabolizes glucose is both a chemoorganotroph and uses oxygen as a terminal electron acceptor) to help populate character matrices and to infer higher-order character states that are not explicitly stated in taxonomic descriptions. Finally, MicrO relates microbiological entities and processes to entities and processes in a large number of other ontologies, including the Gene Ontology (GO), the Phenotypic Quality Ontology (PATO), and the Chemical Entities of Biological Interest (ChEBI) [19–21]. The relationship of classes in MicrO to classes in other ontologies is formalized in a logical, structured, computable way such that MicrO will be able to support future advances in microbial bioinformatics, for example in the automated extraction of text using NLP and integrating microbial characters from different databases or repositories. We anticipate the ontology will provide an important new tool for facilitating the incorporation of massive amount of text descriptions into future generations of biological analysis and computational tools.

## Methods

For the development of MicrO, we took a hybrid top-down and bottom-up approach. For the top-down approach, we used established ontology development principles and practices, such as the use of an upper ontology. In following a bottom-up approach, we used the principles of literary and user warrants [22] and attempted to make the ontology capture the vast diversity of phenotypic character information reported in the prokaryotic taxonomic literature.

### Top-down ontology development

The ontology was constructed using Protege OWL (Web Ontology Language; version 4.3) [23]. It is built upon a Basic Formal Ontology (BFO) foundation, and followed OBO Foundry principles [24, 25]. During the early developmental stages of MicroPIE and MicrO, we created extensive term lists—manually generated lists of terms and synonyms (including variations on spelling) from a large corpus (~1,500) of diverse prokaryotic taxonomic descriptions obtained from the primary scientific literature. We focused on taxonomic descriptions from the Archaea, Cyanobacteria, Mollicutes, Bacteroidetes, and Firmicutes. In this way, we sampled characters from extremophilic chemotrophs, Cyanobacteria (which often have very different taxonomic descriptions and morphological traits), as well as a rich diversity of heterotrophic and chemotrophic, non-pathogenic and pathogenic, species found in the Mollicutes, Bacteroidetes, and Firmicutes. Most non-cyanobacterial descriptions were obtained from the IJSEM, while most cyanobacterial descriptions were sampled from AlgaeBase (an online database of taxonomic descriptions from cyanobacteria and algae) [26].

The term lists were organized hierarchically using categories and subcategories, and this organizational structure is currently used to support MicroPIE. Examples of categories include Colony Morphology, Cell Shape, Metabolic Substrates, Growth Conditions, and Antibiotic Physiology. Each category has a varied number of subcategories, for example, the category Colony Morphology included Colony Shape, Colony Texture, and Colony Color as subcategories. Categories and subcategories in the term lists were matched to higher-level ontology classes and re-organized into candidate qualities, processes, and material entities (including chemical entities and cellular components). These were then used to create the higher-level ontology classes in MicrO, which were then incorporated into the upper level BFO hierarchy.

### Bottom-up ontology development

Lower-level terms in the term list were manually grouped into candidate classes and synonyms in Microsoft Excel. Terms synonymous to existing classes in ontologies in the OBO Foundry were identified using OntoBee [27]. These were imported into MicrO using OntoFox [28].

### Imported classes

Imported classes (Additional file 1: Table S1) were used to provide higher-level classes for the nesting of MicrO-specific classes, to represent microbiological concepts present in other ontologies, and to construct logical axioms for classes in MicrO. Eight classes in BFO were imported, to provide the top-level structure of the ontology. For many ontologies, a relatively small number of lower-level classes were imported. These included BSPO, CHMO, CL, DRON, IAO, NCBI Taxonomy, NDF-RT, OBI, PO, PR, REO, RO, and Uberon (respectively: the Biological Spatial Ontology, Chemical Methods Ontology, Cell Ontology, Drug Ontology, Information Artifact Ontology, NCBI Taxonomy, National Drug File Reference Terminology, Ontology for Biomedical Investigations, Plant Ontology, Protein Ontology, Reagent Ontology, Relations Ontology, and Uber Anatomy Ontology) [29–37]. For other ontologies (ChEBI, GO, PATO), a larger number of higher- and lower-level classes were imported. This was because these classes were used to construct the bulk of the logical axioms in MicrO. Classes from CL, ENVO, and IDO (the Cell Ontology, Environment Ontology, and Infectious Disease Ontology) [38, 39] were imported to help support the future construction of logical axioms as MicrO expands to incorporate new sets of classes (such as pathogenic phenotypes and microbial habitats). For IAO and RO, imported terms were nearly entirely object and datatype properties. These were used to construct logical axioms, and also served as parent classes for new object properties in MicrO.

Because much of microbial diversity lies in the metabolic transformation of chemicals, most of the imported classes were from ChEBI (~6,450 classes). Imported classes included various chemical substances (e.g., ‘lecithin’, ‘bacitracin’, ‘collagen’), roles (e.g., ‘biological pigment’, ‘biomarker’, ‘visual indicator’, ‘reducing agent’), and large numbers of inorganic chemicals, organic chemicals, and mixtures. In addition, we submitted term requests for several hundred microbial-specific compounds to ChEBI, including minerals, antibiotics, dyes/stains, lipids, cell wall constituents, and metabolic substrates and products. These new chemical classes were then imported into MicrO. Finally, a large number of synonyms were added to existing and new chemical classes in ChEBI.

Because phenotypes (size, shape, relationships of cells, cell parts, and colonies) are frequently present in prokaryotic taxonomic descriptions, a large number of imported classes (1,580) came from PATO. Imported classes included quality classes (such as ‘morphology’, ‘size’, ‘shape’, ‘physical quality’ and their children), process quality classes, and increased and decreased quality classes.

Features of prokaryotic cells (*e.g*. vacuoles or flagella) as well as biological processes and enzymatic activities are common in prokaryotic descriptions. Hence, many classes (632) were imported from GO. These included classes involved in prokaryotic cell parts (*e.g*., ‘cell hair’, ‘pilus’, ‘periplasmic flagellum’), biological processes (*e.g*., ‘photorespiration’), enzymatic activities (*e.g*., ‘metalloendopeptidase activity’), and biological responses to various chemicals (*e.g*. ‘response to bile acid’).

One hundred and fifteen classes were imported from Uberon. These included classes associated with anatomical structures and organism substances, which can serve as disease targets for pathogenic microorganisms and as material that is processed to generate chemical entities (e.g., 'brain heart infusion') used in the cultivation of microorganisms.

A handful of classes (22) were imported from OBI, including ‘assay’, various entities involved in microbiological assays such as ‘test tube’, ‘microscope slide’, ‘microscope’, ‘culture medium’ and associated entities such as ‘cultured cell population’ and ‘cultured clonal cell population’. One hundred and five classes were imported from CHMO, and included classes such as ‘evaporation’, ‘grinding’, ‘autoclaving’, and ‘sample heating’. These were used to construct axioms involved in microbiological medium ingredients. Imported classes from BSPO (83) included ‘anatomical margin’, ‘anatomical region’, and ‘anatomical side’ and their respective children, to support creation of logical axioms relating to the spatial relationships of differentiated prokaryotic structures. Classes from CL (284) included ‘native cell’, ‘prokaryotic cell’, ‘eukaryotic cell’, and differentiated red and white blood cells (associated with pathogenic phenotypes and used in microbiological diagnostic assays). Seven classes were imported from PO, these included ‘fruit’, ‘seed’, ‘plant embryo’. These classes were used in the construction of logical axioms for microbiological medium ingredients for MicrO classes such as ‘malt extract’, ‘soya extract’, ‘soy peptone, ‘olive oil’, and ‘filtered tomato juice’. Over 500 classes were imported from NCBI Taxonomy to construct logical axioms for entities and qualities that inhere to particular prokaryotic taxa, and to logically connect culture medium recipes used to cultivate particular prokaryotic taxa.

A large number of classes relevant to microbiological habitats and processes (1,962) were imported from ENVO. Although currently few of these classes are used in logical axioms in the current version of MicrO, their presence will support the future development of MicrO (which will involve the incorporation of microbial habitats). Similarly, microbiologically relevant classes from IDO (81 classes) were imported to support the future incorporation of pathogenic phenotypes into MicrO.

### MicrO-specific classes

If no relevant classes in existing ontologies in the OBO Foundry Library could be identified, the candidate classes were converted into ontology classes, and entered into MicrO. Some classes were derived from information contained in commercial and non-commercial websites outlining microbiological concepts (such as colony morphologies, diagnostic assays, and culture medium recipes) or from scientific publications. In such cases, the definition source (website or publication) was cited. Each class also has a list of synonyms found in the corpus of taxonomic descriptions. Class synonyms were annotated in the ontology as exact synonyms, broad synonyms, or related synonyms using naming conventions developed by GO [40]. Classes under the parent imported class OBI:‘assay’ were created and structured using the conventions used by OBI. Compound class naming followed the ANSI/NISO guidelines [22]. Finally, we made use of the HermiT 1.3.8 and the FaCT++ reasoner in Protege to verify performance of logical axioms.

### Availability

MicrO is available in OWL format as a permanent URL [41] and from the project website [42]. MicrO has been incorporated into the OBO Foundry Library so that other ontologies can import classes and build upon it [43]. The contents of the ontology are available under a CC-BY license [44].

## Results and discussion

### Overview of ontology contents

MicrO (version 1.3, released on March 23, 2016) consists of ~2550 classes (plus thousands of synonyms) derived from text contained in the taxonomic descriptions of diverse prokaryotic taxa that span the archaeal and bacterial domains of life. MicrO incorporates more than 12,000 additional relevant terms from 19 other ontologies in the OBO Foundry Library and these imported terms are connected to MicrO classes using a large number of logical axioms (over 24,130, with 5,446 specific to MicrO). The largest categories of classes in the ontology include assays (enzymatic, metabolic, and phenotypic assays), microbiological culture media and media ingredients, and prokaryotic qualities (including colony morphologies, shapes, and sizes). Other types of classes (such as those describing prokaryotic cell and cell parts) are scattered and nested within GO classes. Finally, a handful of classes in MicrO are scattered in various other parts of the ontology. The large-scale architecture of classes of material entities, processes, and qualities in MicrO, and how they nest in other ontologies, is shown in Additional file 1: Figures S1-S3.

### Prokaryotic chemical entities

A large number of new chemical classes (>750) were entered into ChEBI as a result of MicrO development. New ChEBI classes include minerals (including sulfide minerals), stains/dyes, metabolic substrates, lipids, inorganic chemicals, and antibiotics. In addition, requests were made to add synonyms (188) to existing and new ChEBI classes. Many microbiologically specific chemical mixtures, however, were retained under MicrO. These were categorized into ‘defined inorganic chemical mixture’ (62 classes), ‘undefined inorganic chemical mixture’ (4 classes), ‘defined organic chemical mixture’ (29 classes), and ‘undefined organic chemical mixture’ (121 classes; Additional file 1: Figure S4). Examples of defined inorganic chemical mixtures include ‘trace elements solution SL-6’ and ‘modified MJ synthetic sea water’. Examples of undefined inorganic chemical mixtures, used as ingredients in microbiological culture media, include ‘filtered aged seawater’ and ‘sea salt’. Examples of defined organic chemical mixtures include ‘Balch vitamin solution’, ‘dried bovine hemoglobin’, and ‘hemin solution’. Examples of undefined organic chemical mixtures include ‘clarified rumen fluid’, ‘ox bile salts’, ‘egg yolk oil’, ‘laked rabbit blood’, and ‘inspissated serum’. Additional classes were created for complex mixtures that were produced from hydrous, enzymatic, or chemical extraction of other material entities (*e.g*., ‘yeast extract’, ‘proteose peptone’, ‘casamino acids’, ‘crude oil extract’, and ’casein hydrolysate’).

### Culture media recipes

**Fig. 1:**
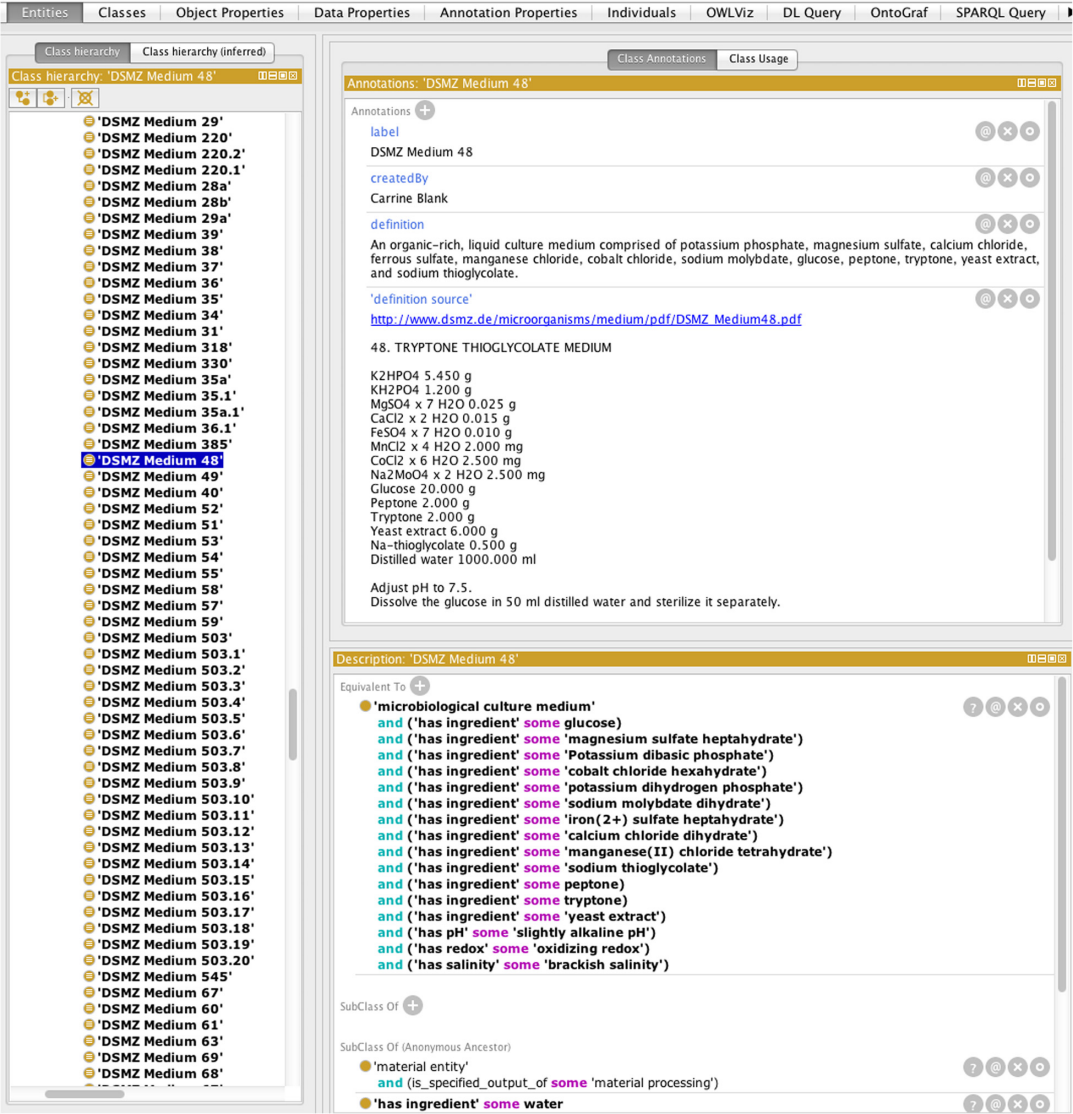
Screen Capture Showing Microbiological Culture Medium Recipes and Logical Axioms Employed. Logical axioms for these classes included the chemical ingredients used to make up the medium in addition to several Value Partitions that described the pH, salinity, and redox of the culture medium (for example: ‘has salinity’ some ‘brackish salinity’)

Microbiological culture media recipes (~910 classes) were included, under the parent class OBI:’culture medium’ (Fig. 1). Annotations include the recipe, the citation or web link to the recipe, and synonyms of the class. Logical axioms included the chemical ingredients used for each medium (connecting MicrO terms to ChEBI terms). Value Partitions were created to categorize different types of culture media. For example, one Value Partition is related to the pH of the medium; whether it was strongly acidic (pH <4), moderately acidic (pH 4–5.5), slightly acidic (pH 5.5–6.5), near neutral pH (pH 6.5–7.5), slightly alkaline (pH 7.5–8.5), moderately alkaline (pH 8.5–10.0), or strongly alkaline (pH >10.0). Another Value Partition related to the salinity of the medium using salinity values that are commonly used in biology; whether it was freshwater (<0.05 % salts), brackish (0.05–3.0 %), marine (3.0–5.0 %), or hypersaline (> 5.0 %). A third Value Partition related to the redox (the oxidation-reduction potential) of the medium; whether it was oxidizing (oxygen or air were present and not containing reducing agents), mildly reducing (containing organosulfides or thiosulfate), or strongly reducing (containing cysteine, glutathione, 2-mercaptoethanol, dithiothreitol, sodium sulfide, hydrogen sulfide, dithionite, or titanium citrate). Covering axioms were put in place for each of the Value Partitions. The logical axioms that were created were designed to facilitate future studies that rely on the logical inference power of the ontology to gain higher-order knowledge of microbial taxa based on the chemical composition of their growth media, such as studies seeking to identify correlations between phylogeny and culture medium chemistry [45]. Finally, the logical axioms put in place can help fill out the knowledge gap of MicroPIE. For instance, taxonomic descriptions will often state the type of media in which an organism is capable of growing. The logical inference power made possible by the ontology allows MicroPIE to immediately compute the chemical conditions under which that particular organism is capable of growing (even if given only the names of the culture medium).

### Assays

A large number of classes (~570) describe microbiological diagnostic assays, under the parent class OBI:‘assay’. Assays include cell staining assays, commercial suites of diagnostic assays (e.g., API microbial ID test kits, Biolog, RapID, and VITEK), salinity, pH and redox assays), a large number of organic carbon metabolism assays (including organic acid alkalinization assays, organic carbon assimilation assays, organic carbon fermentation assays, and organic carbon fermentation/oxidation assays), milk reactivity assays, motility assays, hemadsorption/hemagglutination/hemolysis assays, coagulase assays, growth response assays (including growth response to various antibiotics, inorganic chemicals, and organic chemicals), and finally a large number of specific enzymatic assays (e.g. ‘beta-galactosidase assay’, ‘catalase assay’, ‘lecithinase assay’, ‘pyruvate decarboxylase assay’).

**Fig. 2:**
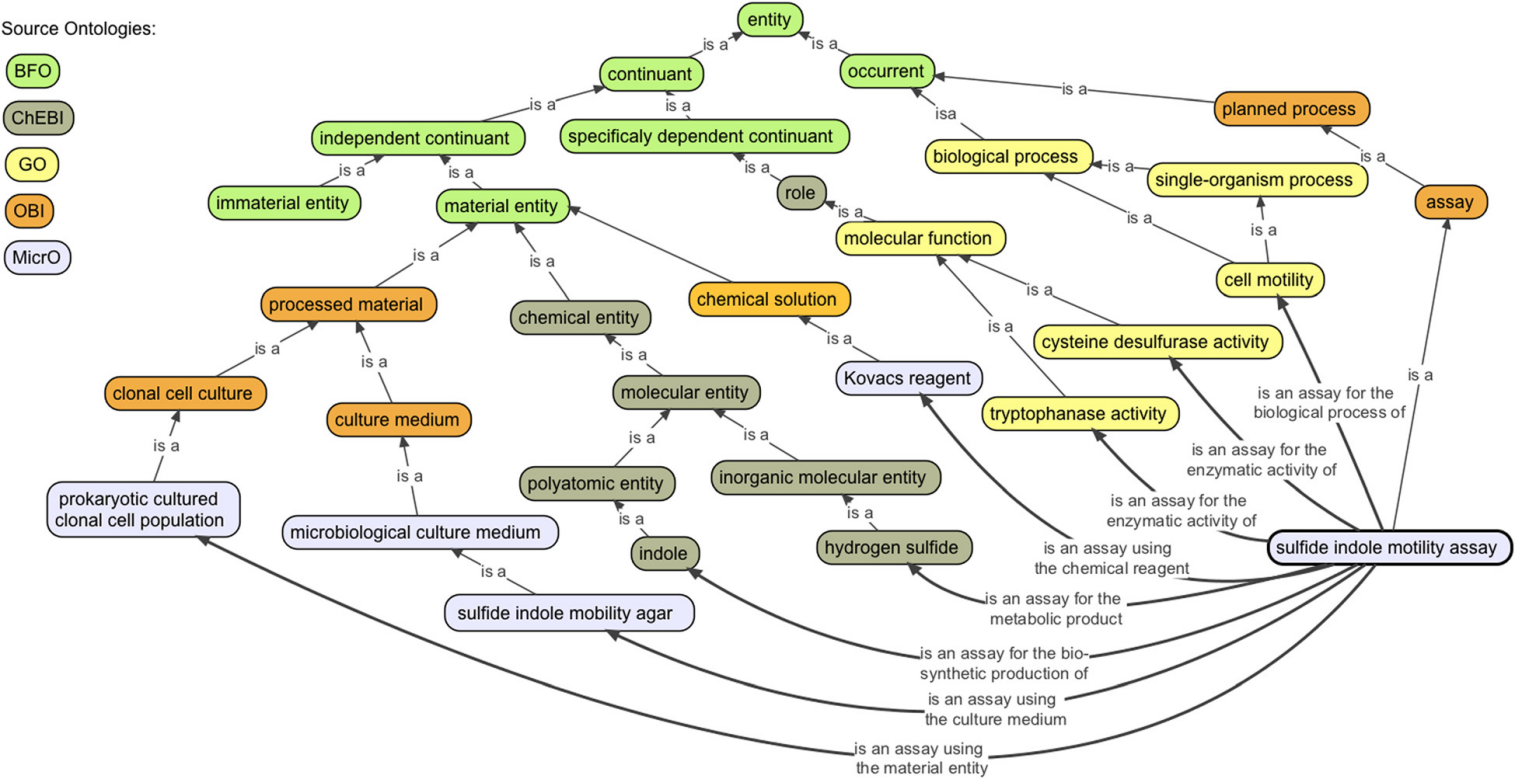
Pattern for Assay Classes. Schematic showing part of the logical design pattern for microbiological diagnostic assays (*e.g.* the class ‘sulfide indole mobility assay’). Multiple logical axioms connect various assay classes to other classes (such as ‘microbiological culture medium’) in other parts of the ontology using object properties (shown with curved, bolded lines)

Assays, with axioms connecting substrates, products, and enzymatic activities were important to have in the ontology, because most prokaryotic taxonomic descriptions describe the outcomes of particular assays performed on the particular isolate being described and logical axioms for this set of classes tended to be more complex. The assays are logically connected to chemical entities (e.g. ‘is an assay for the metabolic product’ some ‘hydrogen sulfide’ and ‘is an assay using the culture medium’ some ‘sulfide indole motility agar’) and processes (e.g., ‘is an assay for the biological process of’ some ‘cell motility’ and ‘is an assay for the enzymatic activity of’ some ‘tryptophanase activity’; Fig. 2 and Additional file 1: Figure S5). Logical axioms also include the enzymatic substrates (some of which are colorimetric compounds, such as ‘5-bromo-4-chloro-3-indolyl beta-D-galactoside’) and products, and the culture medium used to perform the test (e.g., ‘is an assay using the culture medium’ some ‘sulfide indole motility agar’).

Sometimes, taxonomic descriptions will report lists of enzymatic reactions that were tested and provided a positive or negative test result (e.g., positive for valine arylamidase), while other times they will report lists of the substrates hydrolyzed or not hydrolyzed (e.g., L-valine-2-naphthylamide hydrolyzed). The structure of the ontology connects these two concepts and recognizes that they both relate to the same enzymatic trait (in this case, valine arylamidase activity, assayed using the L-valine arylamidase assay). This is accomplished by including the assay substrates (in this case L-valine-2-naphthylamide) as a substrate in the logical axiom for the valine arylamidase assay class.

### Prokaryotic qualities

Several classes (97) were created to describe prokaryotic qualities. These include prokaryotic cell part qualities (such as ‘gas vacuole quality’, ‘thylakoid quality’, ‘Gram stain quality’, and ‘prokaryotic cell wall lysis susceptibility’), prokaryotic cell qualities (such as ‘cell granulation’, ‘cell pigmentation’, ‘cell size quality’, and ‘flagellar quality’), and ‘prokaryotic colony quality’. Classes also included prokaryotic metabolic qualities (‘aerobic’, ‘microaerophilic’, ‘aerotolerant’, ‘obligately aerobic’, ‘photofermentative’, ‘chemolithoautotrophic’, ‘photoorganoheterotrophic’, *etc.*) and prokaryotic physiological qualities (including ‘barophilic’, ‘obligately barophilic’, ‘barotolerant’, and ‘requires magnesium for growth’).

### Prokaryotic cell and cellular components

Many new classes (255) were placed under the parent ‘prokaryotic cell’ including ‘flagellated cell (with subclasses including ‘multiply flagellated’, ‘amphilophotrichous cell’, ‘amphitrichous cell’, ‘lophotrichous cell’, and ‘peritrichous cell’), ‘gas vacuolated cell’, ‘granulated cell’, nanocytes, and ‘pigmented cell’. Classes under ‘morphologically distinct prokaryotic cell’ include ‘bacilloid cell’, ‘cuboidal cell’, ‘pear-shaped cell’, and ‘prosthecate cell’. Classes under ‘prokaryotic differentiated cell’ include ‘hormogonium’, ‘central endospore’, lateral endospore’, ‘subterminal endospore’, ’basal heterocyte’, and ‘terminal heterocyte’. Classes under ‘prokaryotic metabolically differentiated cell’ include ‘autotroph’, ‘obligate aerobe’, and ‘chemoorganoheterotroph’. Classes under ‘prokaryotic physiologically differentiated cell’ include ‘acidophile’, ‘obligate barophile’, thermophile, and ‘facultative halophile’. Classes under ‘differentiated cyanobacterial filament part’ include ‘conical apical cell’, ‘tapered by apical narrowing’, ‘isopolar metameric’, ‘multiseriate filament’, and ‘subterminal meristematic zones’.

Classes (49) were created to describe prokaryotic colonies. The structural organization of classes relating to colonies with distinct morphologies, sizes, and shapes, mirrored the class organization of ‘morphology’, ‘size’, and ‘shape’ in PATO (Additional file 1: Figure S6). This helped to facilitate the construction of logical axioms between classes in MicrO and PATO. For example, under the parent class ‘prokaryotic colony’ were placed the classes ‘morphologically distinct colony’, ‘physically distinct colony’, and ‘colony having distinct process quality’. ‘Morphologically distinct colony’ is logically defined as ‘prokaryotic colony’ and ‘has morphology’ some ‘PATO:morphology’.

MicrO classes of cell parts (~128 classes) include ‘pseudopeptidoglycan-based cell wall’, ‘teichoic acid-based cell wall’, ‘sheath’, and ‘proteinaceous sheath’. Additional prokaryotic cell parts include ‘cyanobacterial filament part’, ‘filament branch’, trichome, ‘heteropolar trichome’, ‘tapered trichome’, ‘isopolar trichome’, ‘trichome part’, ‘apical cell’, ‘basal heterocyte’, ‘medial cell’, ‘necritic cell’, *etc*. Under ‘cyanobacterial filament’, classes include ‘multi-trichomous filament’, ‘multiseriate filament’, ‘biseriate filament’, and ‘uniseriate filament’. Our plan is to submit term requests for relevant classes of cell parts that should belong in GO.

### Prokaryotic biological processes

Finally, 41 classes were created that defined prokaryotic biological processes (lithotrophy, mixotrophy, anaerobic respiration using various electron acceptors and donors). These classes are embedded into GO classes, and may be expanded upon and incorporated into GO in the future. Logical axioms connect these biological processes with chemical entities (e.g. ‘uses electron acceptor’ some ‘nitrate’, ‘uses carbon source’ some ‘organic molecular entity’), other processes (*e.g.,* ‘has part’ some ‘phototrophy’ and ‘has part’ some ‘heterotrophy’), and biological entities (*e.g.,* ‘is prokaryotic metabolic process occurring in’ some ‘mixotroph’).

### Object and datatype properties

In order to connect classes in MicrO to those in external ontologies, we imported object properties from IAO, OBI, RO, and Uberon. We also created ~77 new object and datatype properties to relate microbial-specific classes to one another (Additional file 1: Table S2). Many of the new Object Properties are nested within OBI or RO parent classes. New object properties were assigned definitions and (when possible) domains and ranges.

### Application and future directions

Microbial diversity is vast. Our ontology did not focus on pathogenic phenotypes (such as hosts, target organs, and diseases). These are areas that will need further ontology integration with other existing ontologies (for example, with OMP, the Disease Ontology, Infectious Disease Ontology, the Pathogenic Disease Ontology, and the Human Disease Ontology) [46–48]. MicrO also did not focus on microbial habitats. Development of ENVO is ongoing and the incorporation of microbial habitats into ENVO is a potential fruitful new approach for integrating MicrO with ENVO. Also, there are a number of new prokaryote-focused ontologies in development focusing on microbial metagenomic metadata and microbial habitats/environments (such as MEOWL; Microbial Environments described using OWL; https://github.com/hurwitzlab/meowl). These can be incorporated into MicrO and formal logical axiom linkages added to further increase axiomization of microbial terms. Finally, our ontology did not cover traits associated with microbial eukaryotes.

In the near future, we plan to incorporate MicrO into our developing NLP program (MicroPIE), and in doing so will greatly increase the computing power of MicroPIE. Currently, MicroPIE relies on term lists, which treat each term as an individual entity. MicroPIE cannot determine that the terms ‘rod’, ‘bacillus’, ‘bacilli’, ‘elongated cocci’, and ‘short cylinders’ are all synonyms for the same concept (a bacillus shape). MicrO, with its controlled vocabulary, logical axioms, and annotations including synonyms, can inform NLP programs like MicroPIE that these are indeed the same class, and hence streamline the functionality of the algorithm. The ontology will help MicroPIE recognize that terms such as ‘mixotroph’ and ‘mixotrophic’ all point to the same concept (the ability to carry out process of mixotrophy). The ontology will also reduce confusion in facilitating the identification of synonymous concepts when it comes to the varied reporting of the results of prokaryotic diagnostic assays (as discussed above).

Because of the logical inference power provided by the ontology, MicrO will allow algorithms like MicroPIE to infer new information about a microbial taxon that is not explicitly stated in the taxonomic description. For example, if an organism metabolizes glucose and is photosynthetic, MicrO-enabled MicroPIE can infer that it is a photoorganotroph. If an organism grows at 89 °C, MicrO-enabled MicroPIE can infer that it is a hyperthermophile (given that the logical definition for a hyperthermophile in MicrO constrains an organism’s optimal growth temperature to being above 85 °C). If an organism has akinetes, MicrO-enabled MicroPIE will be able to infer that it is in the Nostocales or Stigonematales (two Orders in the Cyanobacteria). These inferred character states can help to populate cells of a matrix that can be quite sparse when NLP is used to extract literal characters from text.

Additionally, MicrO will be able to support a future generation of bioinformatics capabilities for the microbiological community. For example, because MicrO connects phenotypic information and diagnostic assays with the enzymatic activities in GO, it could be used to support future work aimed at connecting microbial phenotypes with genotypes (*i.e*., the gene content in genomes). Exciting new tools and approaches for connecting phenotypes with genotypes are being developed for metazoans [49–51]. These tools could be adapted and expanded to similarly function with microbial taxa and microbial genomes in the future, given that the field of microbiology now has a rich ontology. In this manner, MicrO could be a useful tool for other researchers in the field of metagenomics and evolution of microbial phenotypic traits.

## Conclusions

MicrO is an ontology of prokaryotic phenotypes and metabolic characters, which also includes classes for microbiological media recipes and diagnostic assays. The ontology uses a controlled vocabulary, detailed annotations, and an extensive set of logical axioms to connect prokaryotic classes (including qualities, processes, assays, and entities) to terms from 19 outside ontologies. By connecting microbial concepts with chemical entities, material entities, biological processes, molecular functions, and qualities from existing ontologies in the OBO Foundry using logical axioms, we intend MicrO to be a powerful new tool which will help push forward progress on the natural language processing of prokaryotic taxonomic descriptions, and make possible new connections between microbial phenotypes and genotypes (i.e. gene content in genomes). Future ontology development will include incorporation of pathogenic phenotypes (such as hosts, target organs, and diseases) and prokaryotic habitats.

## Additional file

Additional file 1: Supplemental Figures (Figures S1-S6) and Tables (Tables S1 and S2). (DOCX 1785 kb)

## Abbreviations

BFO: basic formal ontology
BSPO: biological spatial ontology
ChEBI: chemical entities of biological interest
CHMO: chemical methods ontology
CL: cell ontology
DRON: drug ontology
NDF-RT: national drug file reference terminology
ENVO: environment ontology
GO: gene ontology
IAO: information artifact ontology
IDO: infectious disease ontology
OBI: ontology for biomedical investigations
OMP: ontology of microbial phenotypes
MicroPIE: microbial phenomics information extractor
NLP: natural language processing
OBO: open biomedical ontologies
OWL: web ontology language
PATO: phenotype quality ontology
PO: plant ontology
PR: protein ontology
REO: reagent ontology
RO: relations ontology
Uberon: uber anatomy ontology
