# Supplementary material for: MicrO: an ontology of phenotypic and metabolic characters, assays, and culture media found in prokaryotic taxonomic descriptions
Source: J Biomed Semantics. 2016 Apr 12;7:18. doi: 10.1186/s13326-016-0060-6 (PMC4830071; doi:10.1186/s13326-016-0060-6)

**Additional file 1**

**Table S1.** Imported Classes in MicrO (version 1.1).

| Source ontology | # of Classes | Object Properties | Data Properties | Top level classes | Use in MicrO |
| --- | --- | --- | --- | --- | --- |
| BFO | 8 | 0 | 0 | All classes | Provides high-level organizational structure |
| BSPO | 83 | 10 | 0 | ‘anatomical region’ | To construct logical axioms for classes that describe the relative positioning of differentiated microbial structures |
| ChEBI | 6458 (including temporary terms)^1^ | 8 | 0 | ‘chemical entity’ and ‘role’ | To define composition of microbiological culture media, includes the higher-order structure of classes to aid future logical inference for NLP |
| CHMO | 105 | 1 | 0 | ‘material processing’ | To define classes of microbiological medium ingredients |
| CL | 284 | 0 | 0 | ‘cell’ and ‘native cell’ (including red and white blood cell types and ‘prokaryotic cell’ | To construct logical axioms involving classes involved in assays, and undefined culture media ingredients, used as parent class for prokaryotic cell morphologies and cell parts. |
| DRON | 2 | 0 | 0 | Skim milk | To construct logical axioms for ingredients in microbiological culture media recipes and in assays |
| ENVO | 1962 | 1 | 0 | ‘environmental system’, ‘environmental feature’, ‘environmental material’, ‘environmental condition’ | To construct a small number of logical axioms with classes in MicrO. Will help support future incorporation of microbial habitats. |
| GO | 632 | 0 | 0 | ‘biological process’, ‘cellular component’ and ‘molecular function’ | To construct numerous logical axioms with many classes in MicrO. |
| IAO | 0 | 1 | 1 | ‘has measurement unit label’ and ‘has measurement value’ | To construct logical axioms that involve data properties |
| IDO | 81 | 0 | 0 | ‘host role’, ‘infectious disease’, ‘material entities’ regarding infectious disease, ‘pathogen role’, pathogenic disposition’, protective resistance’, ‘symbiont role’, and ‘toxin disposition’ | Currently not used in axioms in MicrO, but will help support future incorporation of pathogenic phenotypes. |
| NCBI Taxonomy | 564 | 0 | 0 | ‘Archaea’, ‘Bacteria <prokaryote>’, and ‘Eukaryota’ | To construct logical axioms in MicrO involving prokaryotic phenotypic qualities and cell parts. |
| NDF-RT | 36 | 0 | 11 | Chemical Ingredients | To construct logical axioms for ingredients in microbiological culture media recipes and in assays |
| OBI | 22 | 15 | 0 | ‘chemical solution’, ‘processed material’, and ‘planned process’ (including ‘assay’ and ‘material processing’) | To construct logical axioms in MicrO involving assays and culture medium ingredients. Used to nest classes involving culture media and assays.. |
| PATO | 1580 | 0 | 0 | ‘decreased quality’, ‘increased quality’ and ‘quality’ | To construct numerous logical axioms in MicrO involving morphologies of colonies, cells, and cell parts, and the increased concentration of chemicals in media. |
| PO | 17 | 3 | 0 | ‘plant structure’ | To construct logical axioms in MicrO involving culture medium ingredients (which derive from plant ingredients). |
| PR | 3 | 0 | 0 | Amino acid chain | To construct logical axioms in microbiological culture medium ingredients |
| REO | 2 | 2 | 0 | Bovine serum albumin | To construct logical axioms in microbiological culture medium ingredients |
| RO | 0 | 125 | 0 | are all object properties | Object properties are used to construct logical axioms in MicrO, and used as parents for MicrO-specific object properties. |
| Uberon | 115 | 16 | 0 | ‘anatomical entity’ | To construct logical axioms in MicrO involved in colony morphologies, culture medium ingredients, and the spatial relationships of differentiated cells or cell parts. |

^1^Temporary terms, comprised of terms in term requests sent to ChEBI, have been created in MicrO.

**Table S2.** Object and Data Type Properties in MicrO.

| **Object Properties:** | | | |
| --- | --- | --- | --- |
| Label | SubProperty Of | Domain(s) | Range(s) |
| MicrO:contains biological process | BFO:contains process | MicrO:prokaryotic differentiated cell | GO:biological process |
| MicrO:contains prokaryotic metabolic process | MicrO:contains biological process | MicrO:prokaryotic differentiated cell | MicrO:prokaryotic metabolic process |
| MicrO:has measurement |  |  |  |
| MicrO:has angle | BFO:has part |  |  |
| MicrO:has cell wall | BFO:has part | NCBITaxonomy:cellular organisms | GO:external encapsulating structure |
| MicrO:has chemical composition | BFO:has part | BFO:material entity | BFO:material entity |
| MicrO:has ingredient | MicrO:has chemical composition | ChEBI:mixture or OBI:culture medium or OBI:chemical solution | ChEBI:chemical entity |
| MicrO:has measurement unit label | BFO:has part |  |  |
| MicrO:is an assay using the material entity | OBI:has specified input | OBI:assay | BFO:material entity |
| MicrO:is an assay using the cellular organism | MicrO:is an assay using the material entity | OBI:assay | NCBITax:cellular organisms |
| MicrO:is an assay using the chemical reagent | MicrO:is an assay using the material entity | OBI:assay | ChEBI:chemical entity |
| MicrO:is an assay for the metabolic substrate | MicrO:is an assay using the chemical reagent | OBI:assay | ChEBI:chemical entity |
| MicrO:is an assay for the enzymatic substrate | MicrO:is an assay for the metabolic substrate | OBI:assay | ChEBI:chemical entity |
| MicrO:is an assay for the fermentation substrate | MicrO:is an assay for the metabolic substrate | OBI:assay | ChEBI:chemical entity |
| MicrO:is an assay using the culture medium | MicrO:is an assay using the material entity | OBI:assay | MicrO:microbiological culture medium |
| MicrO:is an assay for the biosynthetic production of | OBI:has specified output | OBI:assay | ChEBI:chemical entity |
| MicrO:is an assay for the metabolic product | OBI:has specified output | OBI:assay | ChEBI:chemical entity |
| MicrO:is an assay for the enzymatic product | MicrO:is an assay for the metabolic product | OBI:assay | ChEBI:chemical entity |
| MicrO:is an assay for the fermentation product | MicrO:is an assay for the metabolic product | OBI:assay | ChEBI:chemical entity |
| MicrO:culture medium has quality | RO:has quality | MicrO:microbiological culture medium | PATO:quality |
| MicrO:has pH | MicrO:culture medium has quality | MicrO:microbiological culture medium | MicrO:pH quality |
| MicrO:has redox | MicrO:culture medium has quality | MicrO:microbiological culture medium | MicrO:redox quality |
| MicrO:has salinity | MicrO:culture medium has quality | MicrO:microbiological culture medium | MicrO: salinity quality |
| MicrO:has physical object quality | RO:has quality | GO:cellular component or MicrO:prokaryotic cell or MicrO:prokaryotic colony | PATO:physical object quality |
| MicrO:has morphology | MicrO:has physical object quality | GO:cellular component or MicrO:prokaryotic cell or MicrO:prokaryotic colony | PATO:morphology |
| MicrO:has shape | MicrO:has morphology | GO:cellular component or MicrO:prokaryotic cell or MicrO:prokaryotic colony | PATO:shape |
| MicrO:has size | MicrO:has morphology | GO:cellular component or MicrO:prokaryotic cell or MicrO:prokaryotic colony | PATO:size |
| MicrO:has structure | MicrO:has morphology | GO:cellular component or MicrO:prokaryotic cell or MicrO:prokaryotic colony | PATO:structure |
| MicrO:has texture | MicrO:has morphology | GO:cellular component or MicrO:prokaryotic cell or MicrO:prokaryotic colony | PATO:texture |
| MicrO:has organismal quality | MicrO:has physical object quality | GO:cellular component or MicrO:prokaryotic cell or MicrO:prokaryotic colony | PATO:organismal quality |
| MicrO:has physical quality | MicrO:has physical object quality | GO:cellular component or MicrO:prokaryotic cell or MicrO:prokaryotic colony | PATO:physical quality |
| MicrO:has position | MicrO:has physical quality | GO:cellular component or MicrO:prokaryotic cell or MicrO:prokaryotic colony | PATO:position |
| MicrO:has spatial pattern | MicrO:has position | GO:cellular component or MicrO:prokaryotic cell or MicrO:prokaryotic colony | PATO:spatial pattern |
| MicrO:has symmetry | MicrO:has spatial pattern | GO:cellular component or MicrO:prokaryotic cell or MicrO:prokaryotic colony | PATO:symmetry |
| MicrO:has colony margin symmetry | MicrO:has symmetry | MicrO:prokaryotic colony | PATO:symmetry |
| MicrO:has prokaryotic metabolic quality | RO:has quality | MicrO:prokaryotic metabolically differentiated cell | MicrO:prokaryotic metabolic quality |
| MicrO:has prokaryotic physiological quality | RO:has quality | MicrO: prokaryotic physiologically differentiated cell | MicrO:prokaryotic physiological quality |
| MicrO:has susceptibility towards the chemical |  | CL:prokaryotic cell or GO:cellular component | ChEBI:chemical entity or OBI:chemical solution |
| MicrO:is an assay for |  | OBI:assay |  |
| MicrO:is an assay for the biological process of | MicrO:is an assay for | OBI:assay | GO:biological process |
| MicrO:is an assay for the cellular component | MicrO:is an assay for | OBI:assay | GO:cellular component |
| MicrO:is an assay for the enzymatic activity of | MicrO:is an assay for | OBI:assay | GO:molecular function |
| MicrO:is an assay for the quality | MicrO:is an assay for | OBI:assay | PATO:quality |
| MicrO:is assayed by |  |  | OBI:assay |
| MicrO:is the biological process assayed by | MicrO:is assayed by | GO:biological process | OBI:assay |
| MicrO:is the cellular component assayed by | MicrO:is assayed by | GO:cellular component | OBI:assay |
| MicrO:is the enzymatic activity assayed by | MicrO:is assayed by | GO:molecular function | OBI:assay |
| MicrO:is the quality assayed by | MicrO:is assayed by | PATO:quality | OBI:assay |
| MicrO:is chemical entity derived from |  | ChEBI:chemical entity | BFO:material entity |
| MicrO:is an extract derived from | MicrO:is chemical entity derived from | MicrO:undefined organic chemical mixture | BFO:material entity |
| MicrO:is an acid hydrolytic extract derived from | MicrO:is an extract derived from | MicrO:undefined organic chemical mixture | BFO:material entity |
| MicrO:is an aqueous extract derived from | MicrO:is an extract derived from | MicrO:undefined organic chemical mixture | BFO:material entity |
| MicrO:is an enzymatic hydrolytic extract derived from | MicrO:is an extract derived from | MicrO:undefined organic chemical mixture | BFO:material entity |
| MicrO:is an oleaginous extract derived from | MicrO:is an extract derived from | MicrO:undefined organic chemical mixture | BFO:material entity |
| MicrO:is prokaryotic metabolic process which makes a cell |  | MicrO:prokaryotic metabolic process | MicrO:prokaryotic metabolic quality |
| MicrO:is prokaryotic metabolic quality of a cell carrying out the process |  | MicrO: prokaryotic metabolic quality | MicrO:prokaryotic metabolic process |
| MicrO:is prokaryotic physiological quality of |  | MicrO:prokaryotic physiological quality | MicrO:prokaryotic physiologically differentiated cell |
| MicrO:is prokaryotic metabolic process carried out in the presence of | BFO:occurs in | MicrO:prokaryotic metabolic process | ChEBI:inorganic molecular entity or ChEBI:organic molecular entity or MicrO:sunlight |
| MicrO:is prokaryotic metabolic process occurring in | BFO:occurs in | MicrO:prokaryotic metabolic process | MicrO:prokaryotic metabolically differentiated cell |
| MicrO:is metabolic substrate assayed by | OBI:is specified input of | ChEBI:chemical entity | OBI:assay |
| MicrO:is enzymatic substrate assayed by | MicrO:is metabolic substrate assayed by | ChEBI:chemical entity | OBI:assay |
| MicrO:is fermentation substrate assayed by | MicrO:is metabolic substrate assayed by | ChEBI:chemical entity | OBI:assay |
| MicrO:biosynthetic production is assayed by | OBI:is specified output of | ChEBI:chemical entity | OBI:assay |
| MicrO:is metabolic product assayed by | OBI:is specified output of | ChEBI:chemical entity | OBI:assay |
| MicrO:is enzymatic product assayed by | MicrO:is metabolic product assayed by | ChEBI:chemical entity | OBI:assay |
| MicrO:is fermentation product assayed by | MicrO:is metabolic product assayed by | ChEBI:chemical entity | OBI:assay |
| MicrO:is prokaryotic metabolic quality of | RO:quality of | MicrO:prokaryotic metabolic quality | MicrO:prokaryotic metabolically differentiated cell |
| MicrO:is quality of the culture medium | RO:quality of | PATO:quality | MicrO:microbiological culture medium |
| MicrO:uses chemical entity |  | MicrO:prokaryotic metabolic process or MicrO:prokaryotic metabolically differentiated cell | ChEBI:chemical entity |
| MicrO:uses carbon source | MicrO:uses chemical entity | MicrO:prokaryotic metabolic process or MicrO:prokaryotic metabolically differentiated cell | ChEBI:chemical entity |
| MicrO:uses electron acceptor | MicrO:uses chemical entity | MicrO:prokaryotic metabolic process or MicrO:prokaryotic metabolically differentiated cell | ChEBI:chemical entity |
| MicrO:uses electron donor | MicrO:uses chemical entity | MicrO:prokaryotic metabolic process or MicrO:prokaryotic metabolically differentiated cell | ChEBI:chemical entity |
| MicrO:uses energy source |  | MicrO:prokaryotic metabolic process or MicrO:prokaryotic metabolically differentiated cell | ChEBI:chemical entity or MicrO:sunlight |
| **Data Type Properties:** | | | |
| MicrO:has measurement value |  |  |  |
| MicrO:has cell width |  |  |  |
| MicrO:has pH value |  |  |  |
| MicrO:has sodium chloride percentage |  |  |  |

**Figure S1. Ontology Schema Showing Example Material Entities and Their Is_a Relationships.**


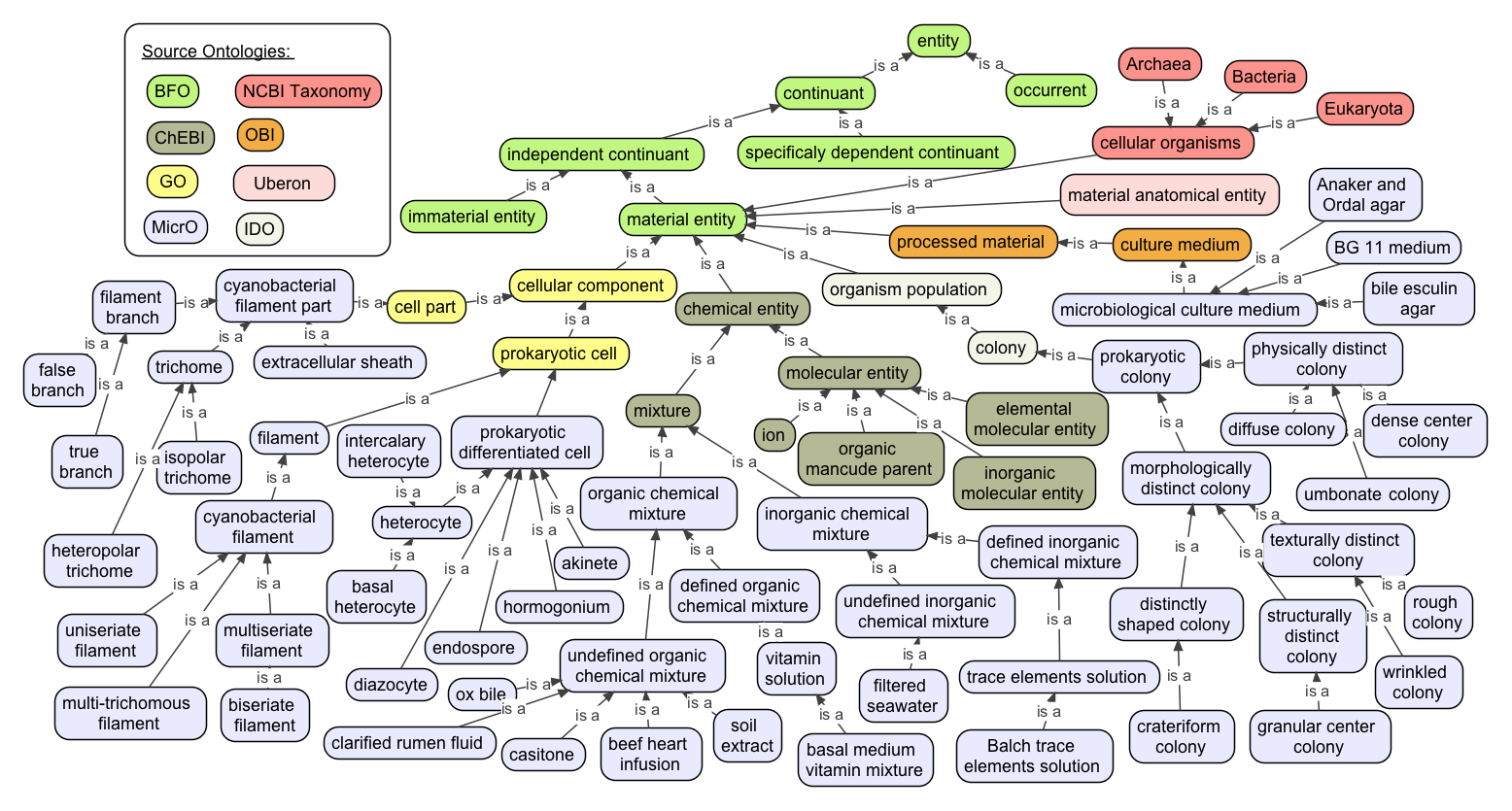


**Figure S2. Ontology Schema Showing Example Processes and Their Is_a Relationships.**


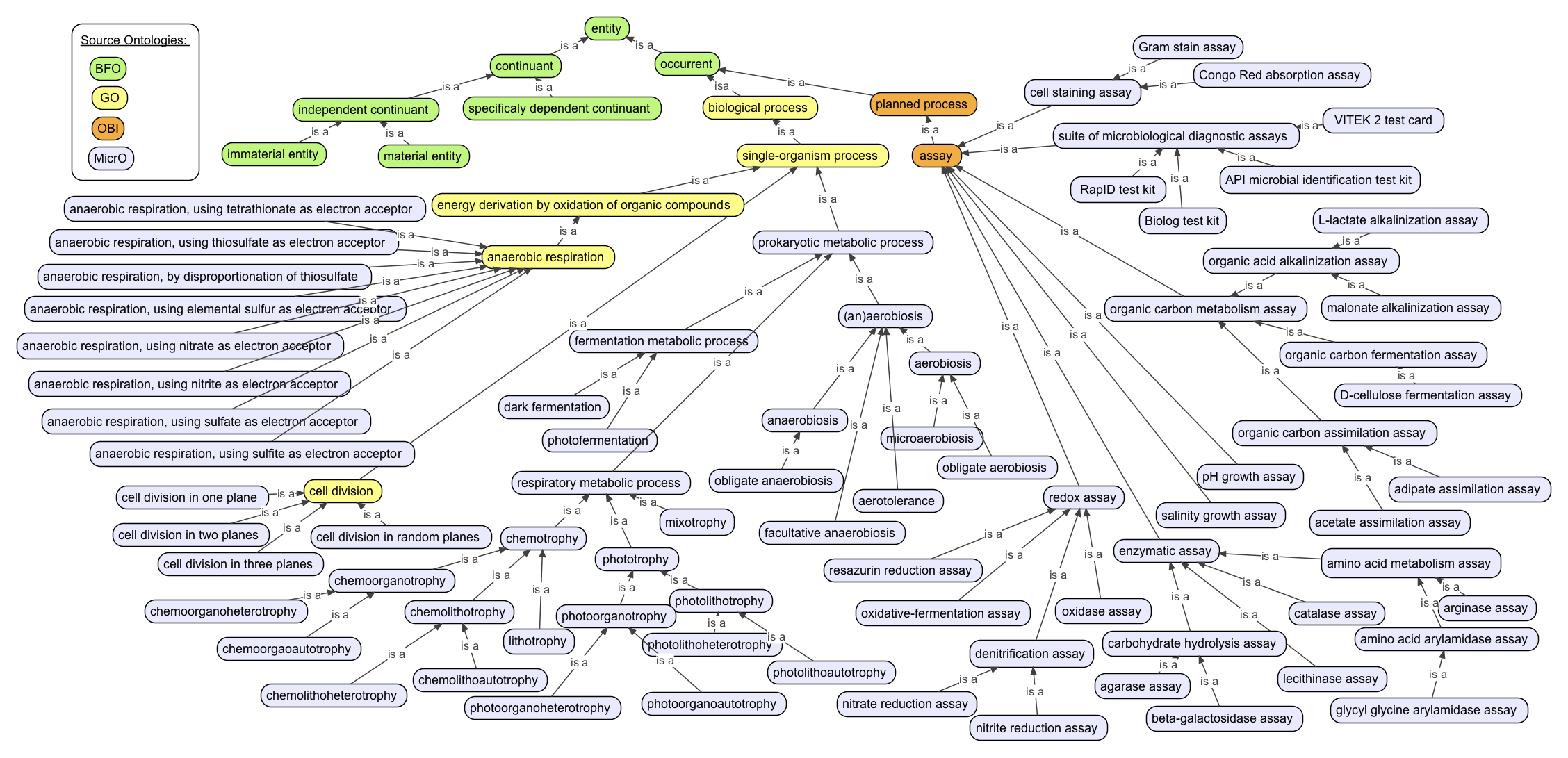


**Figure S3. Ontology Schema Showing Example Qualities and Their Is_a Relationships.**


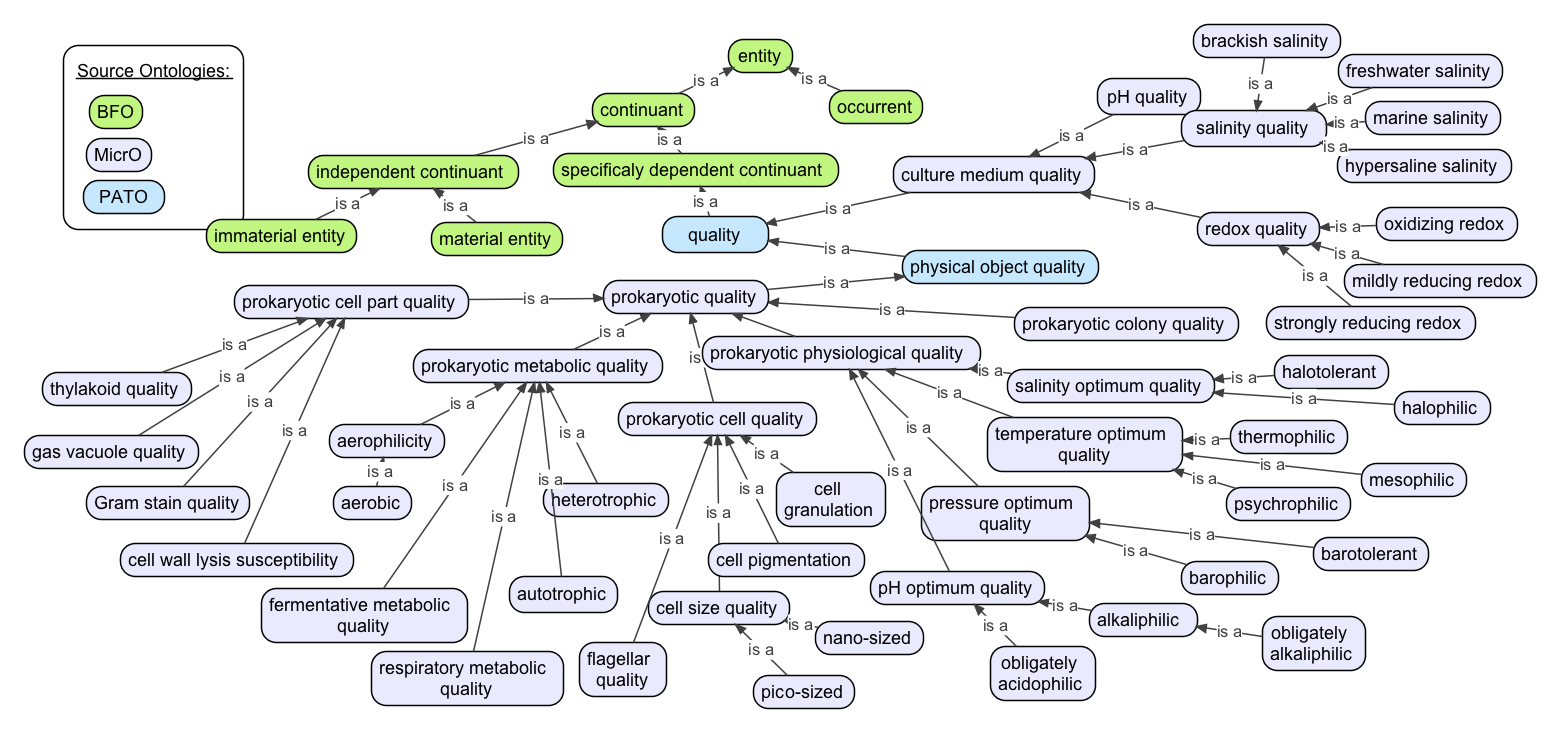


**Figure S4. Protege Screen Capture Showing Class Structure Under the Parent ‘undefined organic chemical mixture’.**


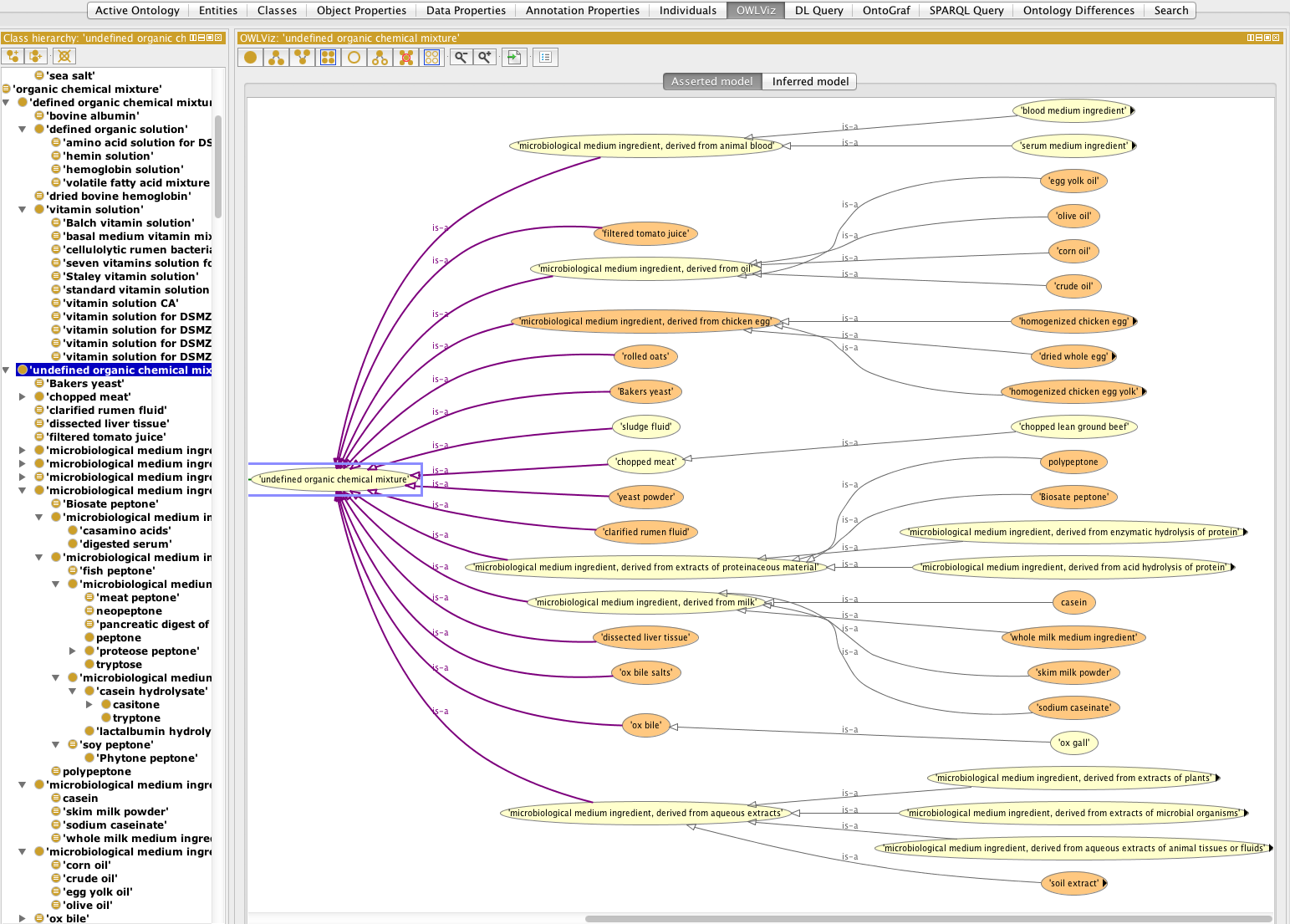


**Figure S5. Screen Capture Showing Logical Axioms For Classes Under the Parent ‘assay’.** Logical axioms included the enzymatic substrates and products (ChEBI classes), the culture medium used in the assay (MicrO classes, not shown in this example), enzymatic activities/molecular functions (GO classes), and biological processes (GO classes, not shown in this example). These classes also contain a number of synonyms for the particular assay, or in this case, related synonyms that refer to the enzymatic substrates used in the assay.


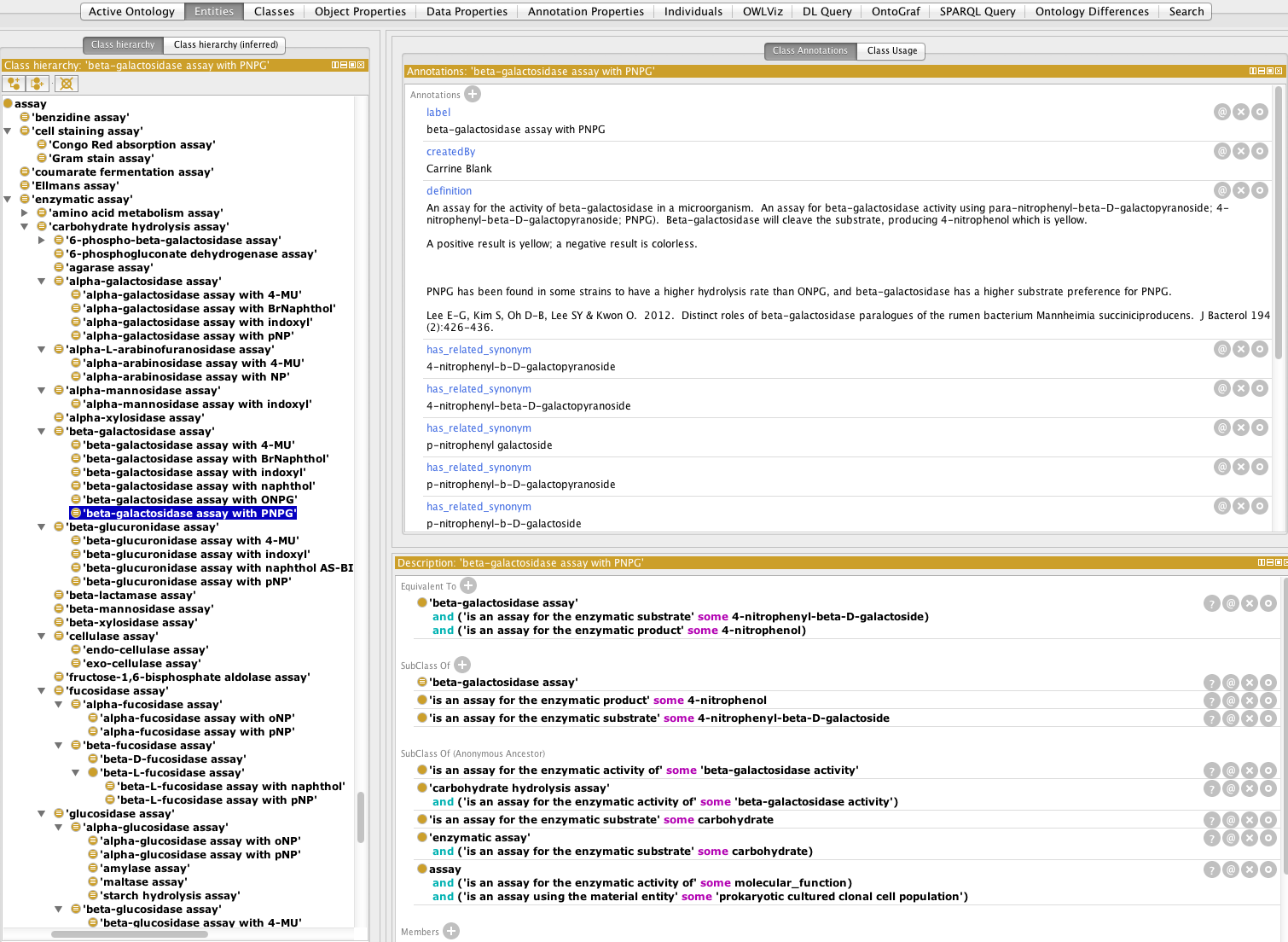


**Figure S6. Screen Capture Showing Logical Axioms For Classes Under the Parent ‘prokaryotic colony’.** Logical axioms connected the MicrO class to the PATO class (in this case PATO:’spindle-shaped’). Broad synonyms were synonyms collected from the taxonomic literature that referred to this particular type of prokaryotic colony.


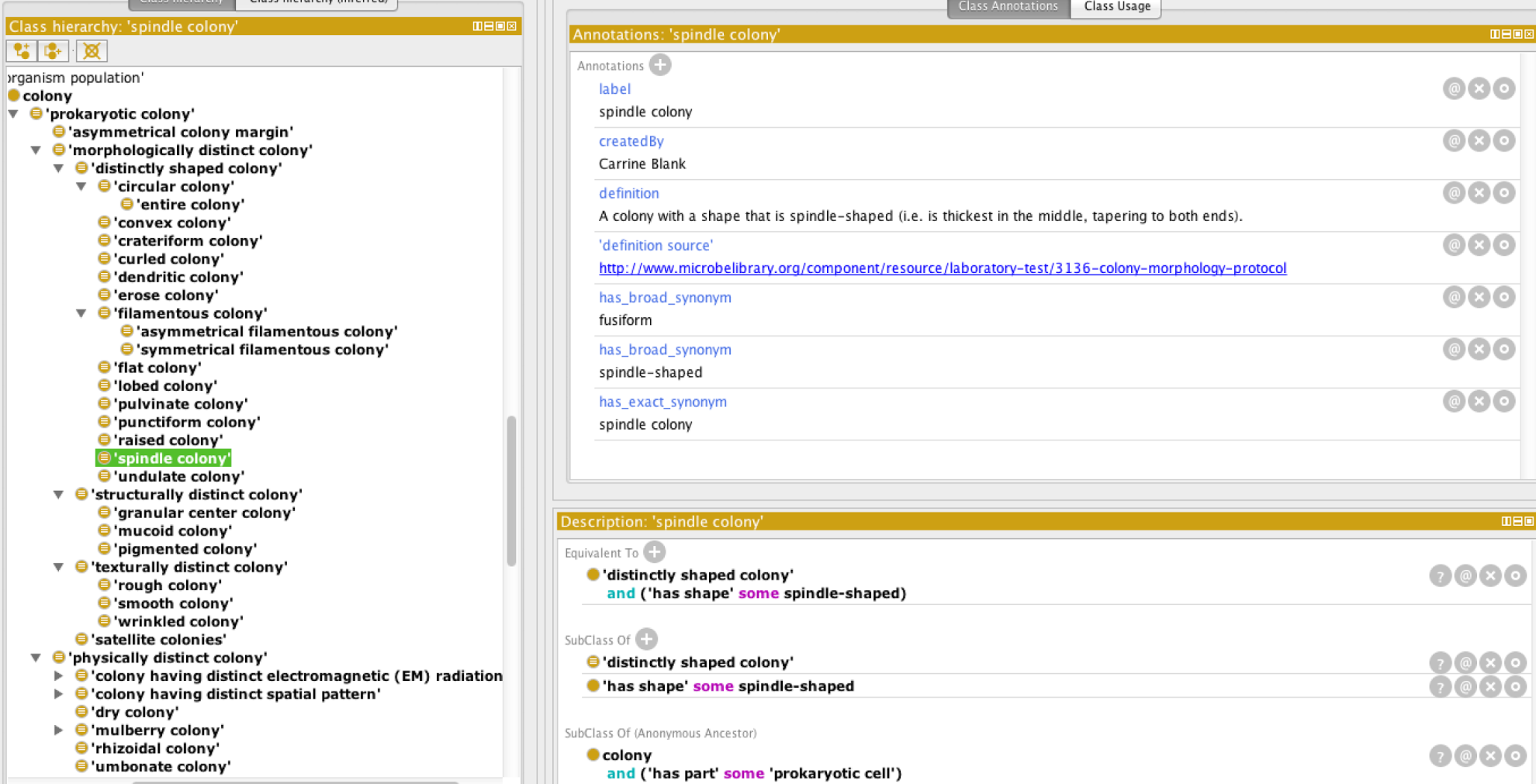

Supplement: Additional file 1: — Supplemental Figures (Figures S1-S6) and Tables (Tables S1 and S2). (DOCX 1785 kb) [file 13326_2016_60_MOESM1_ESM.docx]
